# Reconstructing Face Image from the Thermal Infrared Spectrum to the Visible Spectrum †

**DOI:** 10.3390/s16040568

**Published:** 2016-04-21

**Authors:** Brahmastro Kresnaraman, Daisuke Deguchi, Tomokazu Takahashi, Yoshito Mekada, Ichiro Ide, Hiroshi Murase

**Affiliations:** 1 Graduate School of Information Science, Nagoya University, Furo-cho, Chikusa-ku, Nagoya 464-8601, Japan; ide@is.nagoya-u.ac.jp (I.I.); murase@is.nagoya-u.ac.jp (H.M.); 2 Information Strategy Office, Nagoya University, Furo-cho, Chikusa-ku, Nagoya 464-8601, Japan; ddeguchi@nagoya-u.jp; 3 Faculty of Economics and Information, Gifu Shotoku Gakuen University, 1-38 Naka-Uzura, Gifu 501-6122, Japan; ttakahashi@gifu.shotoku.ac.jp; 4 School of Engineering, Chukyo University, 101 Tokodachi, Kaizu-cho, Toyota 470-0393, Japan; y-mekada@sist.chukyo-u.ac.jp

**Keywords:** face image, thermal infrared, reconstruction, canonical correlation analysis

## Abstract

During the night or in poorly lit areas, thermal cameras are a better choice instead of normal cameras for security surveillance because they do not rely on illumination. A thermal camera is able to detect a person within its view, but identification from only thermal information is not an easy task. The purpose of this paper is to reconstruct the face image of a person from the thermal spectrum to the visible spectrum. After the reconstruction, further image processing can be employed, including identification/recognition. Concretely, we propose a two-step thermal-to-visible-spectrum reconstruction method based on Canonical Correlation Analysis (CCA). The reconstruction is done by utilizing the relationship between images in both thermal infrared and visible spectra obtained by CCA. The whole image is processed in the first step while the second step processes patches in an image. Results show that the proposed method gives satisfying results with the two-step approach and outperforms comparative methods in both quality and recognition evaluations.

## 1. Introduction

Surveillance systems play a critical role in security as they contribute as a means for crime prevention and investigation. In recent years, surveillance systems can be seen in a variety of places, including commercial and even residential buildings. It is important for them to be able to work continuously, day and night. Before one sets up and manages a surveillance system, there are many factors that need to be considered. For example, locations covered by the cameras, level of security that would like to be enforced and the type of the camera itself.

The location that the surveillance system covers is generally divided into two categories: indoors and outdoors. From these two categories, the indoors surveillance system is relatively easier to handle. This is because the illumination conditions can be controlled and there are not many other external factors that can compromise the quality of an indoor image. In contrast, an outdoor surveillance system has many factors that can make the surveillance more difficult. In the outdoors, illumination conditions vary according to the time of day. Weather conditions may also obstruct the quality of captured images.

For the type of camera, cameras that work in the visible spectrum are usually used, which will be referred to as normal cameras from hereon. Normal cameras capture an image depending on illumination, as human eyes do. These cameras are relatively cheap and can be purchased easily. As an example, a basic security surveillance system employs normal cameras to fulfill the role of surveillance.

On the other hand, a thermal camera performs in the thermal infrared spectrum and does not rely on illumination. Instead, it captures infrared radiations that vary depending on the temperature of the object. This capturing process is called thermal imaging. Due to this characteristic, thermal cameras can be a better option over normal cameras for surveillance in nighttime and poorly lit areas.

**Figure 1:**
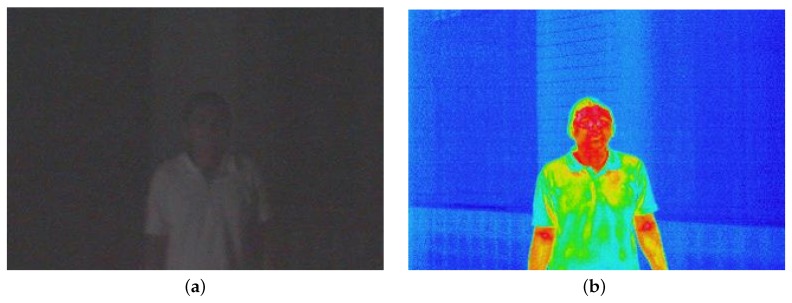
Image examples in different spectra: (**a**) in the visible spectrum; (**b**) in the thermal infrared spectrum.

Figure 1 shows an example of images in both visible and thermal infrared spectra. The presence of a person can be seen clearly in the thermal image, but identifying the person is a different question. The identification of people based only on their thermal information is not an easy task to accomplish.

This paper focuses on the research in reconstructing the thermal face image to the visible spectrum. By doing so, humans can see the face as they usually do: in the visible spectrum. Another advantage of the reconstruction is that further image processing —that is usually done in the visible spectrum— can also be performed. The reconstruction problem can be considered as a subset of the image conversion problem, where the amount of literature is growing steadily.

In another spectrum, namely near-infrared spectrum, there is research on image conversion in the visible spectrum. Among them is research by Chen *et al.* [1], Zhang *et al.* [2], and Goh *et al.* [3]. Although it is also called infrared, the near-infrared spectrum is in fact different from the thermal infrared spectrum. The near-infrared spectrum is the closest to the visible spectrum; therefore, the images captured have similar characteristics to those captured in the visible spectrum. However, due to this similarity, a near-infrared camera also shares some disadvantages of a normal camera, such as the effect of weather changes. In addition, the conversion of a near-infrared image to a visible image is not as difficult as the conversion of a thermal image to a visible image.

Meanwhile, there are only few works available on the modality reconstruction from the thermal spectrum. These works are done by Li *et al.* [4] and Dou *et al.* [5], where both employed a patch-based approach for the reconstruction process, after considering that local facial traits are important for the reconstruction. Li *et al.* [4] utilized Markov Random Fields (MRF) while Dou *et al.* [5] made use of Sophisticated Locally Linear Embedding (LLE) on top of the patch-based method. Therefore, the patch-based approach is considered as the state-of-the-art method in this field. However, the usage of only local facial traits have problems in regards to the facial structure in the visible spectrum. The reason is that this approach ignores the global structure of the face, which, in turn, makes the reconstructed face look choppy and unnatural.

The super-resolution problem, which tries to create a high resolution image from a low resolution one, shares some similarities with this problem. For face images, the process is also commonly known as face hallucination, a term coined by Baker and Kanade [6]. The amount of study in this particular field is plenty in comparison to the previously mentioned fields. For example, works by Liu *et al.* [7] and Ma *et al.* [8] where both of them take a two-step approach, in which the importance of both a global parametric model and a local non-parametric model was shown. The proposed method is inspired by this method, utilizing both the face image as a whole and its local features.

The proposed method also considers the relationship between thermal and visible information of a face. Transforming a face from the thermal spectrum to the visible spectrum can not be done directly, because a factor like changes in temperature can compromise the transformation. However, even when the temperature of a face changes, its thermal pattern will not be affected. In the visible spectrum, the structure of a face also persists, meaning the location of eyes, nose and mouth are relatively fixed. Under these assumptions, the proposed method utilizes Canonical Correlation Analysis (CCA) [9] to learn the relationship between the thermal pattern in the thermal spectrum and the face structure in the visible spectrum. The details of the proposed method are explained in the next section.

The rest of the paper is organized as follows. Section 2 elaborates on the proposed method in detail. Experiments conducted in this research are described in Section 3. Section 4 provides further discussion of the research, and Section 5 concludes this paper.

## 2. Learning-Based Reconstruction Method

**Figure 2:**
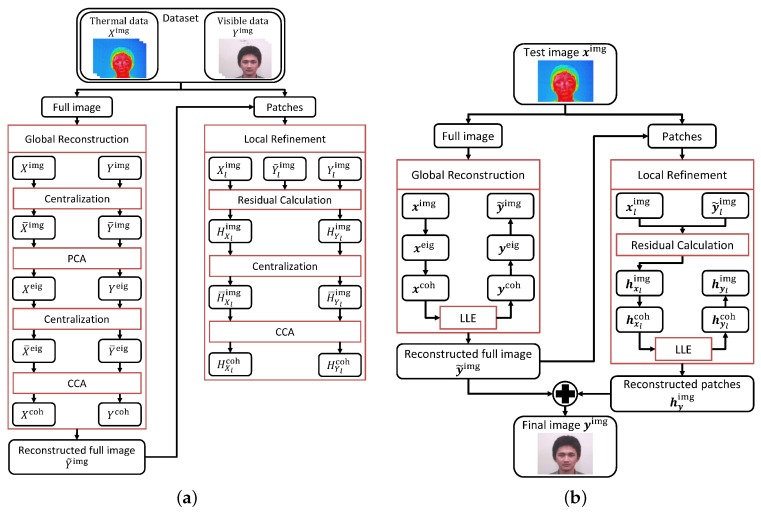
Process flow of the proposed method: (**a**) training process; (**b**) reconstruction process.

As previously mentioned, the method in this research takes a two-step approach, which will be called from hereon as Global Reconstruction and Local Refinement steps, respectively. Each step has their own training and reconstruction processes. Figure 2 shows the flows of the training and the reconstruction processes of the proposed method.

To understand the method more easily, the explanation of the reconstruction method is divided into the two steps; the Global Reconstruction and the Local Refinement. First, the training process of the Global Reconstruction step is described and followed by its reconstruction process. After that, we move on to the training and reconstruction processes of the Local Refinement step.

CCA is employed in the training process of both steps. CCA finds the maximum correlation between the thermal and the visible images, where, in this case, it corresponds to finding the maximum correlation between the thermal pattern and the face structure mentioned previously. Although CCA assumes that the relationship is linear, it can map a thermal information of a face to its counterpart in the visible spectrum.

### 2.1. Global Reconstruction

In this step, a face image is globally reconstructed from the thermal spectrum to the visible spectrum. The reconstructed face image does not possess individuality and details because the reconstruction is performed on the prominent face features. Due to this, the reconstructed face image can be utilized as a base image and further refined in the Local Refinement step. These facial features are extracted with Principal Component Analysis (PCA). The use of CCA in this step is to learn the relationship between the principal components of both the thermal pattern and the face structure from their separate eigenspaces.

#### 2.1.1. Training Process

It is important to point out that in the training process, pairs of face images from both the thermal infrared and the visible spectra are utilized. This also holds true for the Local Refinement step. The notation for thermal data is $X^{\mathrm{space}}=\left(x_{1}^{\mathrm{space}}\;x_{2}^{\mathrm{space}}\;\cdots \;x_{N}^{\mathrm{space}}\right)$ and $Y^{\mathrm{space}}=\left(y_{1}^{\mathrm{space}}\;y_{2}^{\mathrm{space}}\;\cdots \;y_{N}^{\mathrm{space}}\right)$ for visible data, where the superscript “space” indicates which space the data is in with *N* number of training data. In this research, options for “space” are “img”, “eig”, or “coh” for image space, eigenspace, and coherent space, respectively.

Face images in both thermal data and visible data need to be centralized according to Equations (1) and (2). Note that the centralization process is done on each observation n=1,2,⋯,N, creating ${\bar{X}}^{\mathrm{img}}=\left({\bar{x}}_{1}^{\mathrm{img}}\;{\bar{x}}_{2}^{\mathrm{img}}\;\cdots \;{\bar{x}}_{N}^{\mathrm{img}}\right)$ and ${\bar{Y}}^{\mathrm{img}}=\left({\bar{y}}_{1}^{\mathrm{img}}\;{\bar{y}}_{2}^{\mathrm{img}}\;\cdots \;{\bar{y}}_{N}^{\mathrm{img}}\right)$ for thermal and visible images, respectively. $\mu_{X}$ and $\mu_{Y}$ are mean vectors of each spectrum that will be used later:

(1) $${\bar{x}}_{n}^{\mathrm{img}}=x_{n}^{\mathrm{img}}-\mu_{X}$$

(2) $${\bar{y}}_{n}^{\mathrm{img}}=y_{n}^{\mathrm{img}}-\mu_{Y}$$

Training data are then projected onto the eigenspace utilizing projection matrices $P_{X}$ and $P_{Y}$ obtained from applying PCA on the centralized data ${\bar{X}}^{\mathrm{img}}$ and ${\bar{Y}}^{\mathrm{img}}$. This process is shown in Equations (3) and (4). It should be noted that thermal and visible data have their own eigenspaces, represented by $X^{\mathrm{eig}}=\left(x_{1}^{\mathrm{eig}}\;x_{2}^{\mathrm{eig}}\;\cdots \;x_{N}^{\mathrm{eig}}\right)$ for the thermal spectrum and $Y^{\mathrm{eig}}=\left(y_{1}^{\mathrm{eig}}\;y_{2}^{\mathrm{eig}}\;\cdots \;y_{N}^{\mathrm{eig}}\right)$ for the visible spectrum:

(3) $$X^{\mathrm{eig}}=P_{X}{\bar{X}}^{\mathrm{img}}$$

(4) $$Y^{\mathrm{eig}}=P_{Y}{\bar{Y}}^{\mathrm{img}}$$

From their respective eigenspaces, CCA is applied to obtain the correlation between thermal and visible images of the training data. This is done by finding a base for each variable, so that both bases are optimal to the corresponding correlation of the bases. The dimensionality of these new bases is at most equal to the smallest dimensionality of the two variables. Studying these two sets of data with CCA produces two projection matrices, one for each piece of data. These projection matrices are what we need, as the correlation between the projections of the two data is maximized. Details of the CCA can be seen in [10].

Projection matrices $Q_{X}$ and $Q_{Y}$ are obtained from CCA and are used to project the thermal and visible training data from their own eigenspaces onto a coherent space. Before doing so, the centralization process for $X^{\mathrm{eig}}$ and $Y^{\mathrm{eig}}$ is performed as shown in Equations (5) and (6), creating ${\bar{X}}^{\mathrm{eig}}=\left({\bar{x}}_{1}^{\mathrm{eig}}\;{\bar{x}}_{2}^{\mathrm{eig}}\;\cdots \;{\bar{x}}_{N}^{\mathrm{eig}}\right)$ and ${\bar{Y}}^{\mathrm{eig}}=\left({\bar{y}}_{1}^{\mathrm{img}}\;{\bar{y}}_{2}^{\mathrm{img}}\;\cdots \;{\bar{y}}_{N}^{\mathrm{img}}\right)$:

(5) $${\bar{x}}_{n}^{\mathrm{eig}}=x_{n}^{\mathrm{eig}}-\nu_{X}$$

(6) $${\bar{y}}_{n}^{\mathrm{eig}}=y_{n}^{\mathrm{eig}}-\nu_{Y}$$

Similar to Equations (1) and (2), $\nu_{X}$ and $\nu_{Y}$ are mean vectors of each spectrum that will be used later. The projection process is done following that according to Equations (7) and (8):

(7) $$X^{\mathrm{coh}}=Q_{X}^{T}{\bar{X}}^{\mathrm{eig}}$$

(8) $$Y^{\mathrm{coh}}=Q_{Y}^{T}{\bar{Y}}^{\mathrm{eig}}$$

The training data in the coherent space are represented by $X^{\mathrm{coh}}$ and $Y^{\mathrm{coh}}$ for thermal and visible data, respectively. This concludes the training process of the Global Reconstruction step.

#### 2.1.2. Reconstruction Process

The reconstruction process of the Global Reconstruction step involves a new thermal face image $x^{\mathrm{img}}$ that will be reconstructed to a visible face image ${\tilde{y}}^{\mathrm{img}}$. It is important to note that ${\tilde{y}}^{\mathrm{img}}$ represents the globally reconstructed face image and undergoes enhancement later in the Local Refinement step.

The reconstruction begins with the process of projecting a new thermal face image onto the coherent space. Two operations are performed to achieve this, as shown in Equations (9) and (10). Equation (9) shows the process of projecting the face image $x^{\mathrm{img}}$ to the eigenspace while Equation (10) shows the process of projecting the face image $x^{\mathrm{eig}}$ to the coherent space. $x^{\mathrm{coh}}$ is the desired output from these operations, projection of the thermal face image onto the coherent space:

(9) $$x^{\mathrm{eig}}=P_{X}\left(x^{\mathrm{img}}-\mu_{X}\right)$$

(10) $$x^{\mathrm{coh}}=Q_{X}\left(x^{\mathrm{eig}}-\nu_{X}\right)$$

where $\mu_{X}$, $\nu_{X}$, $P_{X}$, and $Q_{X}$ in these equations are obtained from the training process.

Following these operations, the reconstruction is performed in the coherent space by Locally Linear Embedding (LLE) [11]. The LLE is a neighbor-based reconstruction method, meaning that it requires a certain number of neighbors to be able to perform the reconstruction. First, it learns the relationship between the test data and the nearest neighbors in the thermal spectrum. It then calculates the reconstructed test data using that relationship, by applying it to the neighbors’ counterpart in the visible spectrum.

The LLE starts by finding the *K* neighbors of $x^{\mathrm{coh}}$ using the nearest neighbor method. Consider $A_{x}=\left(a_{x}^{1}\;a_{x}^{2}\;\cdots \;a_{x}^{K}\right)$ as the *K* neighbors of $x^{\mathrm{coh}}$. The error function that needs to be minimized in the reconstruction process is shown in Equation (11):

(11) $$\epsilon =|x^{\mathrm{coh}}-\sum_{k=1}^{K}w^{k}a_{x}^{k}|$$

where $w={\left(w^{1},w^{2},\cdots ,w^{K}\right)}^{T}$ is the reconstruction weight vector. To solve this minimization problem, a local gram matrix *G* with $g_{j,k}$ as its element is introduced in Equation (12), where j,k=1,2,⋯,K:

(12) $$g_{j,k}=\left(x^{\mathrm{coh}}-a_{x}^{j}\right)\cdot \left(x^{\mathrm{coh}}-a_{x}^{k}\right)$$

With this, the weight vector ***w*** can be calculated with Equation (13). Here, $g_{j,k}^{-1}$ is an element of $G^{-1}$ (the inverse of matrix *G*). The reconstruction error is minimized by the use of the Lagrange multiplier to enforce $\sum_{k}w^{k}=1$ [12]:

(13) $$w^{k}=\frac{\sum_{j}g_{j,k}^{-1}}{\sum_{j}\sum_{k}g_{j,k}^{-1}}$$

The weight vector ***w*** is then used to estimate the visible data as shown in Equation (14). $A_{y}\;=\;\left(a_{y}^{1}\;a_{y}^{2}\;\cdots \;a_{y}^{K}\right)$ contains *K* neighbors of visible data whose configurations are identical to those of $A_{x}$. This means that the index of $A_{x}$ is referring to the thermal data and the index of $A_{y}$ is referring to its pair, which is the visible data:

(14) $$y^{\mathrm{coh}}=\sum_{k=1}^{K}w^{k}a_{y}^{k}$$

where $y^{\mathrm{coh}}$ is the reconstructed visible data in the coherent space.

The last process in this phase is to project the newly reconstructed visible data back to the image space. Equation (15) with $Q_{Y}^{†}={\left(Q_{Y}Q_{Y}^{T}\right)}^{-1}Q_{Y}$ as the pseudo inverse shows the projection from the coherent space onto the eigenspace, while Equation (16) shows the projection from the eigenspace onto the image space. $\mu^{Y}$ and $\nu^{Y}$ in these equations are averages obtained from Equations (2) and (6):

(15) $$y^{\mathrm{eig}}=Q_{Y}^{†}y^{\mathrm{coh}}+\nu_{Y}$$

(16) $${\tilde{y}}^{\mathrm{img}}=P_{Y}y^{\mathrm{eig}}+\mu_{Y}$$

where ${\tilde{y}}^{\mathrm{img}}$ represents the globally reconstructed visible face image and will be used later in the reconstruction process of the Local Refinement step.

### 2.2. Local Refinement

**Figure 3:**
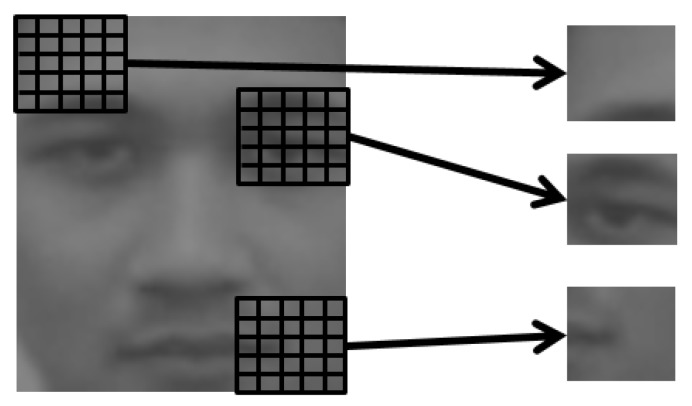
Examples of patches taken from a face image.

The Local Refinement step is where the details of the face are reintroduced, as it was lost in the Global Reconstruction. In order to accomplish this, we employ multiple reconstructions to patches of the globally reconstructed image. As the size of the patch is small, a feature extraction method like PCA is no longer necessary. This ensures the details in the patches are not lost. In this step, CCA learns the relationship of patches from the thermal and the visible spectra directly from the image space. The patches are retrieved by a sliding window that moves through an image and created with overlapping pixel information. Figure 3 shows some visual examples of the patches.

The main idea of the Local Refinement step is to use residual components to perform enhancement/refinement of globally reconstructed data. For that purpose, the training process learns the relationship of residual components in the training data, while the reconstruction process utilizes the residual components for refinement.

#### 2.2.1. Training Process

First, in order to proceed with the training process, it is necessary to have the globally reconstructed images of training data. This is the reason why the reconstruction process of the Global Reconstruction step is explained in advance. The reconstructed training data are represented by ${\tilde{Y}}_{n,l}^{\mathrm{img}}=\left({\tilde{y}}_{1,l}^{\mathrm{img}}\;{\tilde{y}}_{2,l}^{\mathrm{img}}\;\cdots \;{\tilde{y}}_{N,l}^{\mathrm{img}}\right)$, where n=1,2,⋯,N represents an observation with l=1,2,⋯,L as its patch index. The reconstructed data can also be called as globally reconstructed data, referring to the Global Reconstruction step.

After the reconstruction, the difference between each of the reconstructed data ${\tilde{Y}}_{n,l}^{\mathrm{img}}$ and the actual training data $Y_{n,l}^{\mathrm{img}}=\left(y_{1,l}^{\mathrm{img}}\;y_{2,l}^{\mathrm{img}}\;\cdots y_{N,l}^{\mathrm{img}}\right)$ can be calculated per patch, according to Equation (17):

(17) $$h_{y_{n,l}}^{\mathrm{img}}=y_{n,l}^{\mathrm{img}}-{\tilde{y}}_{n,l}$$

where $H_{Y_{l}}^{\mathrm{img}}=\left(h_{y_{1,l}}^{\mathrm{img}}\;h_{y_{2,l}}^{\mathrm{img}}\;\cdots \;h_{y_{N,l}}^{\mathrm{img}}\right)$ is the residual components of visible data. This is also done with the thermal data as shown in Equation (18). The obtained residue contains information from the globally reconstructed image instead of information only from the thermal image:

(18) $$h_{x_{n,l}}^{\mathrm{img}}=x_{n,l}^{\mathrm{img}}-{\tilde{y}}_{n,l}$$

where $H_{X_{l}}^{\mathrm{img}}=\left(h_{x_{1,l}}^{\mathrm{img}}\;h_{x_{2,l}}^{\mathrm{img}}\;\cdots \;h_{x_{N,l}}^{\mathrm{img}}\right)$ is the residual components of the thermal data. Both $H_{X_{l}}^{\mathrm{img}}$ and $H_{Y_{l}}^{\mathrm{img}}$ are used in this step to learn the relationship of those residual information from different spectra. The rest of the training process is similar to those in the Global Reconstruction step without the PCA. As a reminder, the PCA is not performed in this step because the details that were lost in the previous step due to the PCA are going to be reintroduced. Furthermore, since the patch size is already small, feature extraction is not necessary.

In order to carry on with the Local Refinement step, the training patches are centralized according to Equations (19) and (20), creating ${\bar{H}}_{X_{l}}^{\mathrm{img}}=\left({\bar{h}}_{x_{1,l}}^{\mathrm{img}}\;{\bar{h}}_{x_{2,l}}^{\mathrm{img}}\;\cdots \;{\bar{h}}_{x_{N,l}}^{\mathrm{img}}\right)$ and ${\bar{H}}_{Y_{l}}^{\mathrm{img}}=\left({\bar{h}}_{y_{1,l}}^{\mathrm{img}}\;{\bar{h}}_{y_{2,l}}^{\mathrm{img}}\;\cdots \;{\bar{h}}_{y_{N,l}}^{\mathrm{img}}\right)$ and then projected straight onto the coherent space as shown in Equations (21) and (22). $\lambda_{X_{l}}$ and $\lambda_{Y_{l}}$ represent the average of patch *l* in the thermal and the visible data, respectively:

(19) $${\bar{h}}_{x_{n,l}}^{\mathrm{img}}=h_{x_{n,l}}^{\mathrm{img}}-\lambda_{X_{l}}$$

(20) $${\bar{h}}_{y_{n,l}}^{\mathrm{img}}=h_{y_{n,l}}^{\mathrm{img}}-\lambda_{Y_{l}}$$

(21) $$H_{X_{l}}^{\mathrm{coh}}=R_{X_{l}}^{T}{\bar{H}}_{X_{l}}^{\mathrm{img}}$$

(22) $$H_{Y_{l}}^{\mathrm{coh}}=R_{Y_{l}}^{T}{\bar{H}}_{Y_{l}}^{\mathrm{img}}$$

$R_{X_{l}}$ and $R_{Y_{l}}$ are projection matrices for patch *l* in each spectrum obtained from CCA, while the training patches in the coherent space are represented by $H_{X_{l}}^{\mathrm{coh}}$ and $H_{Y_{l}}^{\mathrm{coh}}$. After the projection process, the training process of the Local Refinement step concludes.

#### 2.2.2. Reconstruction Process

The reconstruction process involves reconstructing multiple patches of an image. The reconstructed patches are then combined with the reconstruction results of the Global Reconstruction step. The first operation in this step is calculating the residual value $h_{x}^{\mathrm{img}}$ with:

(23) $$h_{x}^{\mathrm{img}}=x^{\mathrm{img}}-{\tilde{y}}^{\mathrm{img}}$$

where ${\tilde{y}}^{\mathrm{img}}$ is the result of the reconstruction process in the Global Reconstruction step.

The patches are retrieved from $h_{x}^{\mathrm{img}}$, represented as $h_{x_{l}}^{\mathrm{img}}$ where l=1,2,⋯,L is the patch index. These patches are then projected onto the coherent space as:

(24) $$h_{x_{l}}^{\mathrm{coh}}=R_{x_{l}}^{T}\left(h_{x_{l}}^{\mathrm{img}}-\lambda_{X_{l}}\right)$$

The succeeding operation is to use LLE to obtain the reconstructed patches. The minimization problem in this case is:

(25) $$\epsilon =|h_{x_{l}}^{\mathrm{coh}}-\sum_{k=1}^{K}w_{l}^{k}b_{x}^{k}|$$

where $w={\left(w_{l}^{1},w_{l}^{2},\cdots ,w_{l}^{K}\right)}^{T}$ is the reconstruction weight vector we require and $B_{x_{l}}=\left(b_{x_{l}}^{1}b_{x_{l}}^{2}\cdots b_{x_{l}}^{K}\right)$ represents *K* nearest neighbors of $h_{x_{l}}^{\mathrm{coh}}$ at patch *l*.

After we obtained the weight vector, the reconstructed visible patch can be calculated with Equation (26). In this equation, $B_{y_{l}}=\left(b_{y_{l}}^{1}b_{y_{l}}^{2}\cdots b_{y_{l}}^{K}\right)$ represents *K* neighbors whose index is the same with $B_{x_{l}}$. Note that this means the weight vector is different for each patch location:

(26) $$h_{y_{l}}^{\mathrm{coh}}=\sum_{k=1}^{K}w_{l}^{k}b_{Y_{l}}^{k}$$

The reconstructed patches are then projected back onto the image space with:

(27) $$h_{y_{l}}^{\mathrm{img}}=R_{Y_{l}}^{†}h_{y_{l}}^{\mathrm{coh}}+\lambda_{Y_{l}}$$

With the reconstructed patches and the reconstructed image from the previous phase, the final image is created by combining them as:

(28) $$y^{\mathrm{img}}={\tilde{y}}^{\mathrm{img}}+h_{y}^{\mathrm{img}}$$

where $h_{y}^{\mathrm{img}}$ in the equation is the average of the overlapping pixels of the patches $h_{y_{l}}^{\mathrm{img}}$. This concludes the Local Refinement step and also the reconstruction process.

## 3. Experiment

The main purpose of this paper is to reconstruct the face image from the thermal infrared spectrum into the visible spectrum. An experiment was conducted to assess the reconstruction capability of the proposed method by evaluating the produced face images.

We utilize a dataset created for this research which consists of face images of 180 Japanese people (169 males and 11 females) with five subtle variations for each person. The data were taken simultaneously in both thermal infrared and visible spectra. In total, we have 1800 images with 900 images per spectrum. The size of these face images were 56×64 pixels. The images were taken indoors at room temperature and available in both thermal infrared and visible spectra. The camera used for data capture was Avionics’ TVS-500EX (Nippon Avionics Co., Ltd, Tokyo, Japan) [13]. The wavelength that can be captured by the camera ranged from 8–14 μm. The face images were taken at the same time, referred to as pairs, and then underwent preprocessing before they were used.

### 3.1. Experimental Setup

An assumption was made in this experiment that a person’s face variation exists in the training data. To elaborate further, from face variations of a person available, one was used for testing while the others were used for training. This means the test images and training images were not intersected. The experiment here was performed with cross validation.

The evaluation methods of the reconstructed face images were Peak Signal to Noise Ratio (PSNR) [14] and Structural Similarity (SSIM) [15]. Both of these methods compared the reconstructed face with its target/original in the visible spectrum. In other words, the evaluation of the reconstructed face quality. Face recognition of the reconstructed face images was also conducted with the EigenFace method [16].

There are several parameters needed to be considered by the proposed method to perform reconstruction of a face image. Among them are the number of neighbors used for reconstruction in the LLE method, patch size and the pixel displacement of the patch used in the Local Refinement phase. For this experiment, the number of neighbors used was five and the patch size was 9×9 pixels. These parameters were obtained empirically through an experiment described in the next section.

The experiment compares the capability of the proposed method, which is a two-step method, with holistic only and patch-based only methods. The experiment was conducted the same way for all of the methods to guarantee fairness. The parameters used are also the same where it is applicable. A holistic only method means the reconstruction is performed utilizing only the whole image, without the patches. In other words, using only the Global Reconstruction step. This method is labeled as Holistic LLE.

On the other hand, the reconstruction of patch-based only method utilizes the patches of an image. It is important to note that the patch-based method was also the method of choice in both Li *et al.* [4] and Dou *et al.* [5]. The patch-based method is further divided into three, based on their reconstruction algorithm. The first one is Patch-Based LLE, which is similar to the Local Refinement step of the proposed method, but it works directly on the image instead of the residual image. The second one is labeled as Patch-Based 1NN, where the method finds the most similar thermal patch from the training data and reconstructs the face using the visible pair of said patch. This reconstruction method is the most conventional out of all the methods experimented in this paper. The last method is labeled as Patch-Based *k*-NN, a method that averages *k* number of visible patches whose thermal pairs are closest to the input patch, where *k* is the number of neighbors.

### 3.2. Results

**Figure 4:**
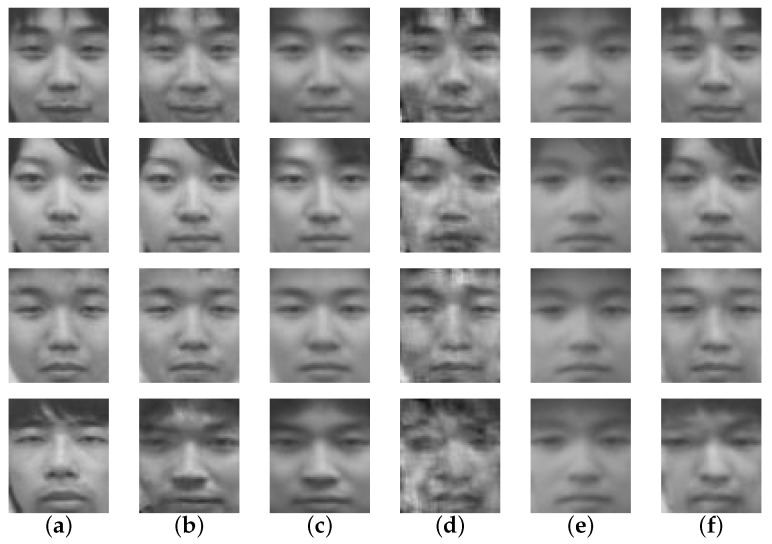
Examples of reconstructed images by various methods. Each row indicates a person and the columns represent images of: (**a**) ground-truth; (**b**) proposed method; (**c**) holistic LLE (Locally Linear Embedding); (**d**) patch-Based LLE; (**e**) patch-Based 1NN (Nearest Neighbor); (**f**) patch-Based *k*-NN.

**Figure 5:**
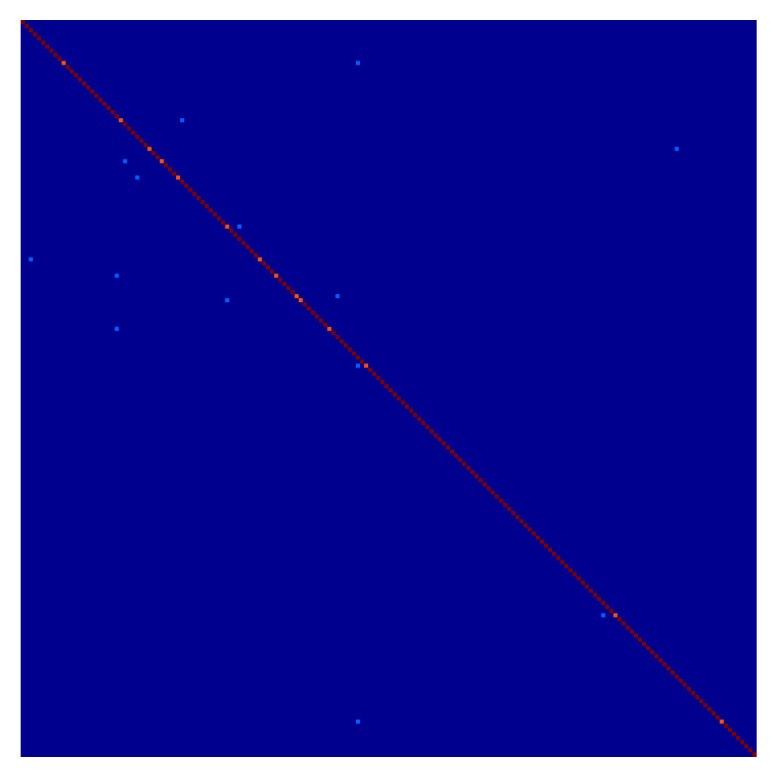
A heat map representation of the confusion matrix of the recognition evaluation. It goes from dark blue to dark red, where the representations of higher values are close to dark red.

**Figure 6:**
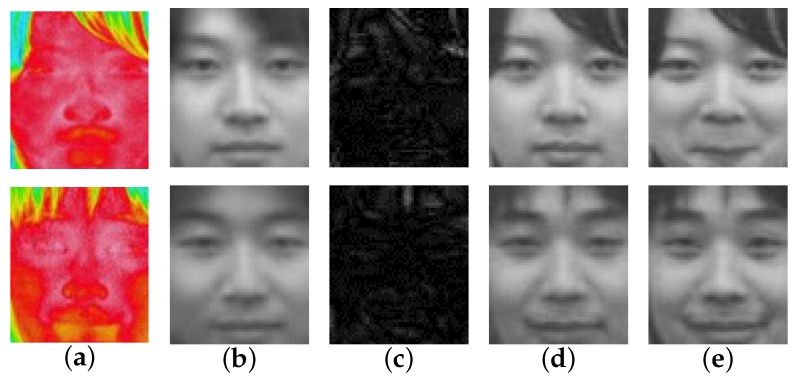
Visual examples in various steps of the proposed method: (**a**) thermal infrared input; (**b**) globally reconstructed images; (**c**) residual images; (**d**) fully reconstructed images; (**e**) ground-truth images.

The evaluation results of the proposed method, the holistic method, and the various patch-based methods can be seen in Table 1. Some examples of the reconstructed face image can be seen in Figure 4. For recognition evaluation, only the recognition rate is shown. Illustrations such as the ROC (Receiver Operating Characteristic) curve are not provided because EigenFace is a nearest neighbor method that has no threshold value. In exchange, a heat map representation of the confusion matrix of the recognition evaluation can be seen in Figure 5. This heat map is specifically of the proposed method. Figure 6 shows visual examples in various steps, also only for the proposed method.

From these results, it can be seen that the proposed method outperformed other comparative methods in all quality evaluations (PSNR and SSIM). The reason is because the proposed method employs both full image reconstruction to create a base face image in the Global Reconstruction step and reintroduces the details of the face in the Local Refinement step. These details are important for these evaluations.

In this research, it is also important to look at the actual reconstructed face images as shown in Figure 4. The reconstruction results of the proposed method resembled its ground-truth closely, where other comparative methods had their own problems in doing so. For Holistic LLE, the reconstructed face images lacked personality and details. In addition, they could be mistaken with someone else. The results of Patch-Based LLE were relatively better than those of the Holistic LLE; they look more similar to the ground-truth. However, there were a lot of artifacts in them. While the Holistic LLE method’s results lack details, the Patch-Based 1NN produces results that have even less details. This is due to the usage of only one visible patch for the reconstruction. On the other hand, the Patch-Based *k*-NN method’s reconstruction results are relatively detailed even though there is still some mis-reconstruction.

For recognition results, the proposed method also outperformed all of the comparative methods. The recognition results of the Holistic LLE almost contend with the proposed method. This is due to the use of EigenFace as the recognition method, where only the prominent features of the face are used to perform the recognition. This means the details of the reconstructed face did not influence the recognition rate, rendering the details reintroduced by the Local Refinement step less effective. As the most conventional of all of the methods, Patch-Based 1NN achieved the lowest performance in all types of evaluations.

There are still failures in these reconstructed images as seen in the last row of Figure 4, among others. All methods struggled to correctly reconstruct this person. A possible reason for this is that there were faces whose thermal patterns were similar to the input, causing LLE to fail in finding the correct neighbors. It is also important to consider that, by using LLE, we assumed that the thermal nearest neighbors calculated from the thermal input have the same geometric relations with the visible counterpart of the input and its neighbors. This is not always the case, however, and this is also a possible reason why the reconstruction can fail. Another reason is that maybe the hair on the upper part of the image hindered the reconstruction process. As a human observer, we can tell that the reconstructed face and the ground-truth are not quite the same person.

It can be concluded that the proposed method takes the best of both holistic only and patch-based only approaches with satisfying results across two evaluation criteria; quality and recognition.

## 4. Discussion

In this section, additional experiments are presented for discussion. As mentioned previously, experiments were done to obtain the optimal set of parameters for the proposed method. Another set of experiments was also conducted to evaluate the proposed method in a more difficult situation.

### 4.1. Finding Optimal Sets of Parameters

The adjustable parameters are patch size and number of neighbors. The options for patch size used in the Local Refinement step were 5×5, 7×7, and 9×9 pixels. The number of neighbors used by the LLE method was selected from either 5, 15, or 30.

The results can be seen in Table 2. Based on these results, the selected set of parameters were five neighbors and 9×9 pixels patch size. This set of parameters achieved the highest results in all evaluation methods. These parameters were used for the comparison experiment between the proposed method, the holistic method, and the patch-based method conducted in Section 3.

### 4.2. Type B: Reconstruction of Unknown Person’s Face Image

In Section 3, an experiment was conducted in the case where the variations of test data were already trained by the proposed method. For further discussion, experiments were done in the opposite case, where an unknown person’s face was reconstructed. This case was more difficult and challenging, because the proposed method did not learn the relationship between the thermal patterns and the visible information of the tested person. For convenience, this experiment will be labeled as Type B. It is also for this reconstruction to be conducted, as the feasibility of the reconstruction needs to be evaluated.

Two types of experiments were conducted in this subsection. The first one was an experiment done to see the performance of the reconstruction for Type B. In the second experiment, we analyzed the effect of increasing the size of training data for Type B.

#### 4.2.1. Performance Evaluation of Type B

The motivation for this experiment is to see the feasibility of reconstructing a face image in this more difficult situation. As mentioned previously, the proposed method did not learn the relationship between the thermal patterns and the visible information of the tested person. Instead, the reconstruction process utilizes the relationship learned from other people in the dataset.

As it is fundamentally different with the previous experiment, the dataset which contains 180 people was divided as follows. Twenty people from the available 180 were separated to be used later in the recognition evaluation. One-hundred-sixty people were divided into 16 groups with 10 people each and cross-validation was performed 16 times. After the face reconstruction of a group was performed, the ground-truths of that group and the 20 people that were excluded earlier were brought together (for a total of 30 people) for the recognition evaluation. This scheme aims to avoid the people used for training in the recognition evaluation, as it raises the possibility of misclassification yet keeps the difficulty of the recognition quite high.

The first experiment was to assess the reconstruction capability of the proposed method for Type B. This experiment is similar to the experiment in the previous section, where we also compare the capability of the proposed method with the holistic only and various patch-based only methods. The results of this experiment can be seen in Table 3.

Based on the results in Table 3, it can be seen that Type B reconstruction is not feasible currently. Improvement of the proposed method is necessary to increase the performance—for example, to learn the relationship between the visible and thermal spectra non-linearly via kernel. Another aspect to consider is increasing the size of the training data, which affects variation of faces available for the reconstruction. Section 4.2.2 provides more detail on this issue.

For quality evaluation, the differences between all of the methods were negligible except for the Patch-Based 1NN. This is contrary to the experiment conducted in the previous section, where the proposed method outperformed all of the comparative methods. For recognition evaluation, however, Patch-Based LLE scored the highest. The reason behind this is highly related to the results of the reconstruction using the whole image. The main problem with performing the reconstruction in this favor is if the reconstructed face was not close to the actual face. When the base face image is far from the truth, reintroducing the details with the Local Refinement step could not improve the results too much. On the other hand, Patch-Based LLE did not make use of the reconstructed base image and reconstructed the face directly in small patches. This method negates the possibility of mis-reconstruction from the whole image.

This explanation also holds true for Patch-Based *k*-NN, which performed the second highest in the recognition evaluation. However, since Patch-Based 1NN only utilizes one visible patch, it is not sufficient to have a satisfactory reconstruction.

**Figure 7:**
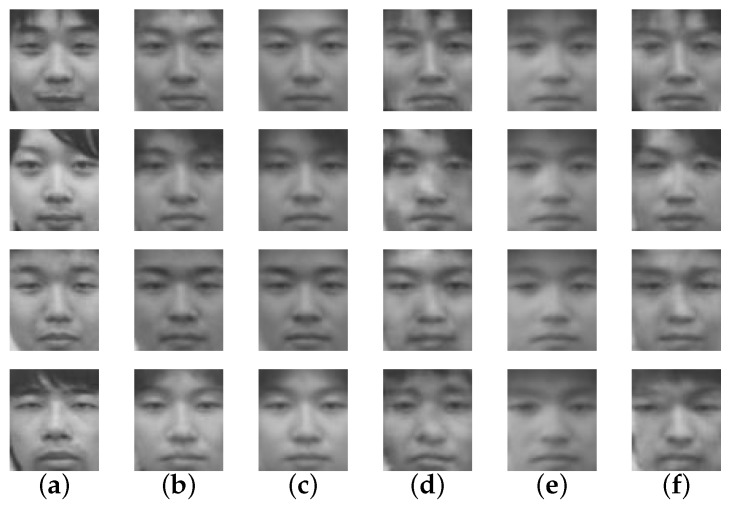
Type B examples of reconstructed images by various methods. Each row indicates a person and the columns represent images of: (**a**) ground-truth; (**b**) proposed method; (**c**) holistic LLE; (**d**) patch-Based LLE; (**e**) patch-Based 1NN; (**f**) patch-Based *k*-NN.

The actual reconstructed faces can be viewed in Figure 7, where we can see that all of the methods struggled to reconstruct the faces. The reconstruction results of the proposed method can be seen as an enhanced version of the holistic method, reinforcing what was mentioned previously. However, all variations of the patch-based approaches produced results that looked unnatural in multiple areas of the face. From these results, we can also conclude that the reconstruction of an unknown person’s face image is difficult.

#### 4.2.2. Type B Reconstruction with Various Sizes of Training Data

The second experiment was conducted to assess the effect of increasing the number of data in the training process. The motivation for this experiment is as follows. As previously mentioned, the proposed method does not know how the unknown thermal face would look like in the visible spectrum. This hampers the proposed method in performing the reconstruction correctly. However, if the number of training data grows, then the available face variations for reconstruction grow as well. This increases the possibility of the proposed method finding a closer match in the reconstruction process.

The way we performed the experiment is as follows. From *x* number of people, 10 people were taken as test data while the rest of them were used for training. After the reconstruction, 20 people outside of the training and test data were combined together with the ground-truths of the test data for a total of 30 people. The reconstructed test data were used as an input for these 30 people recognition evaluation. It is important to note that the same 10 people were used for various numbers of *x*.

The results of this experiment are shown in Table 4. Even though the overall results were still low, the increasing values of PSNR and recognition rate can be seen whereas SSIM values are consistent. This shows that the existence of more variations in the training data helped the reconstruction process. In theory, a very large amount of training data can produce a satisfactory result of reconstructing an unknown person’s face. Having said that, to state the number of data needed is very difficult.

**Figure 8:**
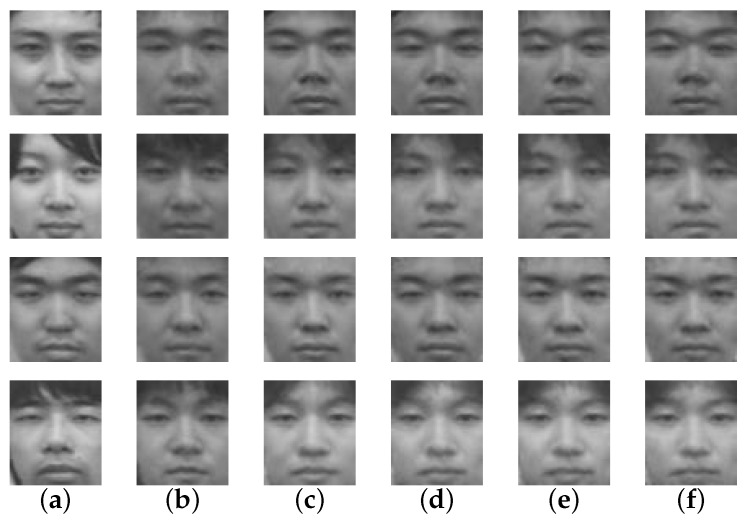
Type B examples of reconstructed images with various number of training data. Each row indicates a person and the columns represent: (**a**) ground-truth images; (**b**) reconstructed images from 40 people’s training data; (**c**) reconstructed images from 70 people’s training data; (**d**) reconstructed images from 100 people’s training data; (**e**) reconstructed images from 130 people’s training data; (**f**) reconstructed images from 160 people’s training data.

The actual reconstructed face images are shown in Figure 8, where the proposed method failed to reconstruct the face satisfactorily. Although the reconstructed faces do not resemble the ground-truths and we can tell that they are not the same person, as the number of the training data increases, changes of the reconstructed faces could be observed. The most significant changes could be observed when the training data increased from 40 persons to 70 persons. Further than that, only small changes were observed.

## 5. Conclusions

This research attempted to reconstruct a face image from the thermal infrared spectrum to the visible spectrum. In order to achieve this, we proposed a two-step reconstruction method. The first step is referred as the Global Reconstruction, where the reconstruction is performed on the whole image. The second one is referred to as the Local Refinement, where patches of the image are reconstructed.

The method we proposed utilizes CCA in the training process in order to understand the relationship between the thermal and the visible images. For the reconstruction of the face image, the proposed method exploits the relationship between the nearest neighbors and the input to reconstruct the face image.

Experiment was done to evaluate the reconstruction capability of the proposed method. Results showed that the proposed method produces high scores in all evaluations and outperforms other comparative methods.

This paper also provided discussion on the reconstruction of an unknown person’s thermal face image, labeled as Type B. The proposed method struggles to perform the reconstruction because there is no information of the face in the visible spectrum. This proves the difficulty of the task and warrants further research in the field. A possible way to solve this is to learn the relationship between the visible and the thermal spectra non-linearly via kernel. The effect of increasing the size of the training data should also be taken into account, because it should increase the variety of faces available, which, in turn, improves the chances of LLE to reconstruct a face image closer to the target. Therefore, the expansion of the dataset both in size and variations (inclusion of other races) is included in our future work.

Another future work is related to the recognition evaluation, where only a qualitative evaluation with the EigenFace method was conducted. Additionally, an experiment can also be conducted to evaluate the reconstruction results by human subjects.

## Figures and Tables

**Table 1:** Comparison of the proposed method with the holistic method and various patch-based methods (Number of neighbors: 5, Patch size: 9×9 pixels). SE represents the standard error.

Method	PSNR (SE) (dB)	SSIM (SE)	Recog. Rate (%)
Proposed	**33.11** (3.69)	**0.95** (0.05)	**98.44**
Holistic LLE	27.04 (1.81)	0.85 (0.05)	98.33
Patch-Based LLE	29.21 (4.09)	0.92 (0.06)	87.33
Patch-Based 1NN	19.47 (1.78)	0.73 (0.06)	1.45
Patch-Based *k*-NN	25.38 (3.41)	0.88 (0.07)	63.78

**Table 2:** Evaluation of various patch sizes with various numbers of neighbors. SE represents the standard error.

# of Neighbors	Patch Size (Pixels)	PSNR (SE) (dB)	SSIM (SE)	Recog. Rate (%)
5	5×5	31.40 (3.20)	0.93 (0.05)	98.33
	7×7	32.57 (3.57)	0.94 (0.05)	98.11
	9×9	**33.11** (3.69)	**0.95** (0.05)	**98.44**
15	5×5	29.88 (2.71)	0.90 (0.06)	97.78
	7×7	31.66 (3.28)	0.93 (0.05)	97.67
	9×9	32.41 (3.50)	0.94 (0.05)	97.78
30	5×5	29.03 (2.48)	0.89 (0.05)	97.56
	7×7	30.31 (2.88)	0.92 (0.05)	97.56
	9×9	31.31 (3.20)	0.93 (0.05)	97.67

**Table 3:** Type B: Comparison of the proposed method with the holistic method and various patch-based methods (Number of neighbors: 5, patch size: 9×9 pixels). SE represents the standard error.

Method	PSNR (SE) (dB)	SSIM (SE)	Recog. Rate (%)
Proposed	19.36 (3.11)	**0.70** (0.13)	12.25
Holistic LLE	19.39 (3.11)	**0.70** (0.13)	11.38
Patch-Based LLE	**19.46** (2.40)	0.69 (0.09)	**23.13**
Patch-Based 1NN	18.05 (1.64)	0.65 (0.07)	3.13
Patch-Based *k*-NN	19.26 (2.41)	0.69 (0.09)	19.88

**Table 4:** Type B: Evaluation of different numbers of training data (Number of neighbors: 5, patch size: 9×9 pixels). SE represents the standard error.

# of People	PSNR (SE) (dB)	SSIM (SE)	Recog. Rate (%)
40	18.74 (2.82)	0.72 (0.09)	4.00
70	19.63 (2.68)	0.74 (0.07)	6.00
100	19.64 (2.89)	0.72 (0.10)	10.00
130	19.80 (2.84)	0.73 (0.10)	6.00
160	20.26 (2.52)	0.73 (0.09)	14.00

## Abbreviations

The following abbreviations are used in this manuscript:

CCA: Canonical Correlation Analysis
LLE: Locally Linear Embedding
MRF: Markov Random Field
NN: Nearest Neighbor
PCA: Principal Component Analysis
PSNR: Peak Signal to Noise Ratio
ROC: Receiver Operating Characteristic
SSIM: Structural Similarity
